# Coach‐Perpetrated Interpersonal Violence: Witnessing, Perceived Harmfulness and the Role of Coaching Motivational Climate

**DOI:** 10.1002/ejsc.70113

**Published:** 2026-01-01

**Authors:** Stiliani “Ani” Chroni, Mary Hassandra, Helena Verhelle, Antonis Alexopoulos, Juan de Dios Benítez‐Sillero, Juan Calmaestra, Per Øystein Hansen, Renzo Kerr‐Cumbo, Sergio Lara‐Bercial, Alexander Navarro, Miguel Nery, Chiara Nicolini, Thiago Santos, Eivind Å. Skille, Sara Vivirito, Tine Vertommen

**Affiliations:** ^1^ Section of Sport and Physical Education University of Inland Norway Elverum Norway; ^2^ Department of PE & Sports Sciences University of Thessaly Trikala Greece; ^3^ Safeguarding Sport & Society Centre of Expertise Care and Well‐being Thomas More University of Applied Sciences Antwerp Belgium; ^4^ European University Cyprus Nicosia Cyprus; ^5^ Universidad de Córdoba Córdoba Spain; ^6^ Sport Coaching Europe Paola Malta; ^7^ MCAST Institute of Community Services Paola Malta; ^8^ Leeds Beckett University Leeds UK; ^9^ International Council of Coaching Excellence Leeds UK; ^10^ Faculdade de Motricidade Humana Universidade de Lisboa Lisbon Portugal; ^11^ CEIPES ETS International Centre for the Promotion of Education and Development Palermo Italy; ^12^ Faculdade de Desporto Universidade do Porto Porto Portugal; ^13^ Department of Movement and Sports Sciences, Faculty of Medicine and Health Sciences Ghent University Ghent Belgium

**Keywords:** harmfulness, interpersonal violence, mediterranean region, motivational climate, sport coach

## Abstract

Coach‐perpetrated interpersonal violence can pose significant risks to athletes' development as well as psychological, physical and social well‐being worldwide. This study examined the perceived harmfulness of witnessed coach‐perpetrated interpersonal violence behaviours in the North Mediterranean region, alongside any associations with coaching climates (empowering and disempowering). Data were collected from 494 active coaches across Cyprus, Greece, Italy, Malta, Spain and Portugal through an online questionnaire where they reported witnessing and perceived harm of psychological, physical, instrumental and sexual violence, as well as their coaching climates. The analysis showed psychological violence as the most frequently witnessed form and physical violence being perceived as the most harmful one. An empowering coaching climate, characterised by autonomy support and positive reinforcement, correlated positively with higher perceived harm, especially for psychological and instrumental violence. Conversely, a disempowering climate, marked by control and punitive behaviours, correlated with lower perceived harm. Gender, coach education and professional status were found to influence coaches' perceptions, highlighting that cultural and structural complexities have a role towards interpersonal violence tolerance. The study underscores the critical need for culturally tailored safe sport initiatives, mandatory training of coaches in safe coaching behaviours and practices and proactive safeguarding measures to mitigate interpersonal violence across diverse sporting contexts. Culturally informed interventions need to challenge the normalisation of violence in coaching and encourage empowering climates that place athletes in the centre and prioritise their welfare.

## Highlights

- Psychological violence is the most frequently witnessed form of interpersonal violence by coaches in the North Mediterranean region, whereas it is perceived as least harmful compared to physical and sexual violence.
- Empowering coaching climates are associated with higher recognition of the harm caused by interpersonal violence behaviours in the region, whereas disempowering climates are associated with lower recognition of such harm.
- Gender, professional status and coach education appear to have an effect on the coaches' perceptions of interpersonal violence, emphasising the need for comprehensive, culturally informed training programmes.

## 1 Introduction

Interpersonal violence (IV) in sport is a global issue, affecting athletes at all levels and across sports disciplines (Tuakli‐Wosornu et al. 2024). IV is defined as ‘the intentional use of physical force or power, threatened or actual, against oneself, another person, or against a group or community, that either results in, or has a high likelihood of resulting in, injury, death, psychological harm, maldevelopment or deprivation’ (Butler et al. 2022, 2–3). Recognising the gravity of IV in sport, the International Olympic Committee (IOC) has issued three consensus statements over the years to raise awareness, educate stakeholders and guide the development of protective measures. The first statement primarily addressed sexual harassment and abuse in sport, highlighting the importance of intervention (IOC Medical Commission Expert Panel 2007). The second expanded the focus to psychological violence, physical violence and neglect (Mountjoy et al. 2016), whereas the most recent statement presented a socio‐ecological model of interpersonal violence in sport and emphasised the need for remedial actions and athlete protection mechanisms (Tuakli‐Wosornu et al. 2024). To address IV in sport, *safe sport* and *safeguarding* emerged as critical frameworks in sport governance. According to the newest IOC statement, safe sport describes the ‘physically and psychologically safe and supportive athletic environment where athletes can thrive and experience the full benefits of sport participation’ (p. 1323), and safeguarding is construed as the proactive and holistic measures necessary for protecting athletes and responding effectively to IV concerns (Tuakli‐Wosornu et al. 2024).

At the same time, participation in sport is widely associated with important benefits for children, youth and adults (Bailey 2006; Eime et al. 2013). When environments are safe and supportive, sport can promote physical health, psychological well‐being, social connection and the development of life skills such as teamwork, discipline and resilience (Bailey 2006; Eime et al. 2013). Recognising this dual potential, sport as both a context for thriving and as a context where interpersonal violence can occur, reinforces the importance of understanding how specific features of the coaching environment may either protect athletes or expose them to harm (Tuakli‐Wosornu et al. 2024).

Despite global and regional initiatives, like the IOC Games‐time Framework (International Olympic Committee, n.d.) or European Union Work Plan for Sport 2024–2027 (European Union 2024), aiming to prevent and appropriately respond to harassment, abuse and violence in sport, substantial variations exist across countries on policy implementation and cultural acceptance of safeguarding measures. A global scoping review revealed wide‐ranging prevalence rates: psychological violence (21%–79%), physical violence (4%–66%), sexual violence (0.5%–78%) and neglect (27%–69%) (Tuakli‐Wosornu et al. 2024). Although some nations have made significant strides, others remain stagnant due to individual, organisational and cultural barriers (Kerr et al. 2019; Tuakli‐Wosornu et al. 2024).

In the European Union (EU), coaches are recognised as primary actors in fostering safe sport environments and member states have been mandated to equip their coaches with essential knowledge, skills and competencies that safeguard athletes (Council of the European Union 2017, 2020). However, recent evidence shows that many coaches themselves lack clarity on what constitutes interpersonal violence and call for more structured training to address it (Gillard et al. 2025). Further, coaches do not operate in isolation—they function within specific cultural and institutional contexts that influence their attitudes and behaviours (Jones and Corsby 2015), including religion, family and community dynamics which are particularly salient in Mediterranean settings (Papaefstathiou 2014). Existing research has associated the prevalence of IV with coaching styles; for instance, authoritarian coaching behaviours were a stronger predictor of sexual harassment experiences of athletes than coach's gender (Sand et al. 2011; Schyvinck et al. 2024). To contribute towards the understanding of IV in sport contexts, this study focused on the North Mediterranean region, where safe sport policies are absent, underdeveloped or inconsistently implemented (Chroni and Kavoura 2022; Chroni and Papaefstathiou 2014; Hartill et al. 2024). Papaefstathiou (2014) described the Cypriot sporting culture as one shaped by close‐knit community ties, high interpersonal trust and tactile coaching practices, which can make safeguarding both sensitive and complex. In this context, where the family's responsibility is emphasised and the state intervenes little, safeguarding measures risk generating moral panic and undermining trust further. In Greece, Chroni and Kavoura (2022) highlighted how pressures to ‘keep the home intact’ and strong cultural deference to authority silence athletes, even when harm is happening. These insights further underscore that institutional mandates for coach training may underappreciate deeply rooted cultural dynamics of the Mediterranean sport contexts. In Italy, Hartill et al. (2024) findings highlight that IV is often normalised, perceived as part of sport and goes largely unreported, reflecting a cultural climate of performance pressures and deference to authority.

### 1.1 Coaching for Safe Sport

IV in sport has been the focus of research for some time. Earlier studies were interested in the coach–athlete relationship and coaches as perpetrators (e.g., Nielsen 2001; Tomlinson and Yorganci 1997), whereas recent findings reveal peer perpetrated IV as highly prevalent (e.g., Hartill et al. 2024; Vertommen et al. 2017). Coaches hold a critical role in establishing a climate that mitigates IV within their team/club (see Jowett et al. 2023). Duda (2013) developed the empowering coaching framework as a practical intervention promoting athlete‐centred coaching. The framework features goal setting, positive engagement and reinforcement as critical components of a safe and supportive environment. By creating an environment that prioritises respect, empowerment and athlete well‐being, coaches can actively contribute to safe sport and foster positive relationships within their teams (Duda and Appleton 2016).

The concept of coaching climate has been linked to safe sport (e.g., Birr et al. 2023; Greither and Ohlert 2023; Ohlert et al. 2022). Recent empirical work from Greither and Ohlert (2023) found that disempowering climates predicted psychological violence, which in turn mediated negative mental health outcomes. Earlier, Ohlert et al. (2022) reported that athletes exposed to sexual violence perceived lower empowering and higher disempowering climates. Together, they suggest that minimising disempowering behaviours and promoting empowering climates may reduce athletes' risk of violence. Similarly, Birr et al. (2023) conducted a scoping review on the coaching climate, identifying key strategies to promote safe sport, such as emphasising athlete autonomy, focusing on mastery rather than performance and fostering positive relationships. Duda et al. (2017) bridged theory and practice by highlighting that youth sport environments with empowering coaching climates foster ethical coaching, positive social interactions and athlete well‐being. Their research highlights the need for coach education, peer mentoring and self‐assessment tools to help coaches refine their motivational strategies.

Though the connection between coaching climate and safe sport is established, in the Mediterranean region, it remains underexplored. In Southern Europe, like in other regions, cultural and social norms play a crucial role in shaping coaching behaviours, often reinforcing hierarchical authority and discipline. Studies from Spain, Greece and Portugal offer insights into the complex interactions between coaching climate and athlete experiences in Mediterranean sport cultures. Castillo‐Jiménez et al. (2022) found that an empowering coaching climate enhanced athletes' psychological well‐being, motivation and long‐term sport participation in Spain. Similarly, Krommidas et al. (2016) found that empowering coaching was positively linked to enjoyment and quality of life in youth soccer players in Greece. In Portugal, Santos et al. (2013) found that some elite coaches create an illusion of empowerment while maintaining strong hierarchical control over athletes, reflecting a cultural disconnect between perceived and actual empowerment. These findings highlight how cultural values, like respect for authority, collectivism and performance‐driven coaching, can impact safe sport initiatives both positively and negatively.

The present study builds on existing literature by providing a culturally specific examination of IV in coaching within the North Mediterranean region. We investigated how the coaching motivational climate associates with witnessing and perceived harmfulness of IV behaviours. By analysing data from coaches in Cyprus, Greece, Italy, Malta, Spain and Portugal, this research offers insights into the interplay between coaching approaches and IV perceptions. The study (i) examined the witnessing of psychological, physical, instrumental and sexual violence perpetrated by coaches; (ii) assessed how coaches perceived the harm caused by IV in six North Mediterranean countries; and (iii) analysed the relationship between the coaching climate and the witnessing and perceived harmfulness of IV behaviours exhibited by coaches. This study's unique contribution lies in its context‐specific analysis of the coaching motivational climate within a region where safe sport policies remain fragmented and sparse. By bridging research with practical applications, this work aims to support evidence‐based policy development and coach training programmes that promote ethical, athlete‐centred coaching practices.

## 2 Method

### 2.1 Participants

The participants were 494 active sport coaches from Cyprus, Greece, Italy, Malta, Spain and Portugal. To partake they had to meet two criteria: being over 18 years of age and be an active coach during the data collection period. Most of them were men (65.4%), 34.3% were women and 0.2% identified with another gender identity. Their mean age was 36 years old (SD = 12.01; *r* = 18–76). Table 1 presents gender and age demographics per country.

**TABLE 1:** Participating coaches' gender and age representation per country.

	Cyprus	Greece	Italy	Malta	Portugal	Spain
	*n* = 85	*n* = 133	*n* = 64	*n* = 75	*n* = 66	*n =* 71
Age *M*(SD)	36.67 (10.93)	34.23 (13.11)	35.51 (10.76)	37.03 (12.74)	39.08 (10.75)	32.78 (12.18)
Gender *n*(%)						
Man	68 (80%)	61 (46%)	38 (59%)	51 (68%)	49 (74%)	56 (79%)
Woman	17 (20%)	72 (54%)	25 (39%)	24 (32%)	17 (26%)	15 (21%)
Other	0 (0%)	0 (0%)	1 (2%)	0 (0%)	0 (0%)	0 (0%)

In total, they represented 44 different sports. Among these sports, football was the most popular (19.4%), followed by swimming (5.9%), gymnastics (4.9%), tennis (4.9%) and basketball (4.5%). Most respondents coached only one sport (65%), more than half (58%) reported coaching both male and female athletes, and several (38.7%) worked with multiple age groups (e.g., children, youth and adults). Table 2 presents detailed information on the participants coaching experiences, professional status, coaching certification and sport background as athletes.

**TABLE 2:** Participating coaches' coaching experience and status, and athletic background.

Coach experience and background		*n* (%)
Gender of participants they coached	Women	62 (12%)
	Men	146 (30%)
	Both genders	286 (58%)
Age of participants they coached	Younger than 12	257 (52%)
	Older than 12	301 (61%)
	Adults	188 (38%)
Coaching participants with disabilities	Yes	61 (13%)
	No	428 (87%)
Competition level they coached	Local	194 (39%)
	Provincial/regional	95 (19%)
	National	124 (25%)
	International/national team	81 (16%)
Professional status as a coach	Paid full time	100 (20%)
	Paid part time	254 (51%)
	Volunteer with expenses paid	55 (11%)
	Volunteer with expenses not paid	85 (17%)
Coaching education certification	Level 1 (basic)	139 (28%)
*Note*: In most countries, Level 1 coaches train beginners and children, Level 2 youth teams and amateur athletes and Level 3 (and up) national‐level teams, elite athletes or professional clubs.	Level 2 (intermediate)	101 (20%)
	Level 3 (advanced)	128 (26%)
	Higher than level 3	54 (11%)
	Other coaching education	21 (4%)
	No coaching certification	67 (14%)
Years of experience as a coach	Less than 1 year	57 (12%)
	Between 1–5 years	149 (31%)
	Between 6–10 years	93 (20%)
	Between 11–20 years	96 (20%)
	Between 21–30 years	59 (12%)
	More than 30 years	21 (4%)
Own experience as an athlete	Yes, in same sport	425 (86%)
	Yes, in other sport	53 (11%)
	No own athlete experience	16 (3%)
Years involved as an athlete	Less than 1 year	2 (0%)
	Between 1–5 years	58 (12%)
	Between 6–10 years	126 (27%)
	Between 11–20 years	199 (43%)
	Between 21–30 years	68 (15%)
	More than 30 years	13 (3%)
Level of involvement as an athlete	Local	120 (25%)
	Provincial/regional	88 (19%)
	National	141 (30%)
	International	127 (26%)

Among the respondents, 70% engaged professionally as paid coaches (full‐ or part‐time) and 30% as volunteers (with or without compensation or expense reimbursement). Concerning their coaching experience, 10% were early career coaches who had been coaching for 1 year only. Among the respondents, the largest group (31%) reported having between one and 5 years of coaching experience. With regard to coach education, the majority (86%) held some level of coaching certification ranging from basic to advanced. Looking at their sport background, 97% had been athletes in the sport they were coaching or a different one, of which 55% had trained and competed at the national and international level.

### 2.2 Measures

To measure the witnessing and perceived harmfulness of psychological, physical, instrumental and sexual violence behaviours, we used items from the coaches' scale of the violence towards athletes questionnaire (VTAQ; Parent et al. 2019). A group of experts with knowledge of sport and cultures in the participating countries, safe sport and IV measurement first reviewed all original VTAQ coach items independently and then convened to agree on a subset. The aim was to retain items that (a) captured core forms of coach‐perpetrated IV that are likely to be observable in everyday coaching practice; (b) could be translated and understood equivalently across Greek, Italian, Portuguese, Spanish and English; and (c) would not be so explicit or taboo in some national contexts that they might prompt shame, denial or early survey dropout. In this process, we removed several items depicting highly explicit sexual acts or extreme physical threats which, in these Mediterranean cultures, are rarely discussed openly and were judged likely to elicit nonresponse or socially desirable responding. Examples of excluded items are ‘touched an athlete's genitals with one's mouth (fellatio, cunnilingus), or hands (masturbation), or rubbed oneself on athlete's genitals’, ‘choked or strangled an athlete’, ‘threatened to abandon, harm an athlete, or harm someone or something they love’. Sixteen items were selected that still covered psychological, physical, instrumental and sexual violence while maximising cultural acceptability and response quality. For each item, coaches answer two questions: (i) ‘Which of the following coaching behaviours do you consider harmful?’ using a four‐point scale ranging from ‘not harmful at all’ (1) to ‘totally harmful’ (4), and (ii) ‘Have you ever witnessed this behaviour?’ with ‘yes, no, or maybe’. Examples of the items are ‘Hitting an athlete with the hand (example: smack, slap)’ (physical); ‘Shouting insults, humiliating or making fun of an athlete’ (psychological); ‘Forcing or asking an athlete to train when injured, when they had contrary medical advice’ (instrumental); and ‘Watching an athlete undress’ (sexual). The 16 items showed good internal consistency on all four forms of IV measured (*⍺*_*physical*_ = 0.782; *⍺*_*psychological*_ = 0.840; *⍺*_*instrumental*_ = 0.782; *⍺*_*sexual*_ = 0.822). In line with our focus on the content and perceptions of core IV behaviours, the four IV types were treated as composite indices based on the adapted subscales. We did not conduct a new confirmatory factor analysis (CFA) of this abbreviated, culturally adapted version of the VTAQ and we return to this as a measurement limitation below.

To increase the probability of participants answering all survey questions, we did not use the full 34‐item empowering and disempowering motivational climate questionnaire (EDMCQ‐C) for coaches (Appleton et al. 2016). Instead, for the purposes of this study, we selected items with the highest factor loading per motivational dimension (autonomy support, social support, task‐involving, ego‐involving, controlling coaching) to measure the empowering and disempowering facets of the motivational climate fostered by the participating coaches. We used 12 items to measure empowering climate (e.g., ‘I try to make sure athletes feel good when they try their best’) and six for disempowering climate (e.g., ‘I yell at athletes for messing up’). Answers were given on a five‐point scale ranging from strongly disagree (1) to strongly agree (5). The internal consistency Cronbach's alpha for the empowering scale was 0.86 and the disempowering 0.60.

### 2.3 Procedures

Approval for the research protocol was obtained for every country's ethics review committee with the exception of Malta which lacks an ethics review agency (national or institutional) and was covered under the approval obtained from the academic institution that led the project. An online survey was developed and administered using the QuestionPro survey software. Respondents could only proceed with the survey questions upon meeting the two inclusion criteria and completing a consent page explaining the purpose and processes of the study, participation being voluntary, how data would be treated anonymously and confidentially and participation could be paused and/or terminated at any point. The measuring instruments were translated into the native languages of the participating countries (Greek, Italian, Portuguese and Spanish) using back‐to‐back translation (Brislin 1970) to ensure linguistic and conceptual equivalence. Maltese coaches used the English version. Due to the sensitivity of the topic, native researchers of participating countries further reviewed the translations for accuracy and clarity with respect to their cultures. As such, small adaptations were made to ensure wording would not cause discomfort.

Every North Mediterranean country partner was responsible to recruit native coaches. The data were collected between January and March 2023, with the aim to reach a minimum of 50 participants per country. A total of 1253 potential participants accessed the online questionnaire. From them, 320 did not meet the inclusion criteria, 648 completed only the demographic section and 17 more were excluded due to not belonging to the participating countries. The significant number of participants that dropped out of the study after completing the socio‐demographic section might be due to the sensitive, possibly distressing, nature of the questions regarding interpersonal violence. This pattern of attrition is consistent with previous research indicating that topics involving abuse, violence or moral transgressions may provoke discomfort, fear of disclosure or concerns about confidentiality, often leading to noncompletion (Tourangeau and Yan 2007). The final sample consisted of 494 coaches of whom 382 completed the full questionnaire and 112 partially completed the measurements of witnessed IV behaviours, perceived harmfulness of these and/or motivational climate. As such, the *n* is reported in all calculations presented in the results.

### 2.4 Statistical Analyses

Descriptive statistics were calculated to ascertain the characteristics of the sample and the studied variables. Chi‐square tests of independence were conducted to examine potential associations between subgroups [gender (man/woman), coaching level (local, regional level/national, international level), professional status (paid/voluntary) and coaching qualifications (holding or not a certification)] and the likelihood of witnessing (yes, no, maybe) each of the 16 IV behaviours in sport. Adjusted standardised residuals were examined to locate the cells in which observed frequencies significantly differed from expected values. In addition, independent *t*‐tests were used to explore differences between the abovementioned subgroups in the dependent variables of IV perceived harmfulness and motivational climate. Prior to this analysis, the assumption of equal variance was tested using Levene's test. When the assumption of equal variances was violated, a Welch's test was used instead of an independent *t*‐test. The effect size was determined using Cohen's categorisation by way of general guidance: *d* < 0.20 indicates a trivial effect, 0.20 ≤ *d* ≤ 0.49 a small effect, 0.50 ≤ *d* ≤ 0.79 a medium effect and *d* ≥ 0.80 a large effect (Téllez et al. 2015). Lastly, we conducted correlations and four multiple regression analyses to assess the unique contributions, strength and direction of empowering and disempowering coaching climates on the perceived harmfulness of various violence behaviours and to examine their explanatory power on coaches' perceptions beyond simple correlations. The SPSS version 27 statistical software package was used to analyse the data.

## 3 Results

### 3.1 Descriptives

More than half of the coaches reported having witnessed all psychological violence behaviours captured by the items, with prevalence ranging from 53% to 70%. Specifically, the items ‘criticising an athlete excessively’ and ‘shouting insults’ scored above 60%, indicating a high prevalence of psychological violence. The number of coaches witnessing sexual violence against an athlete was generally lower, with all items scoring below 20%, except for the item ‘watching an athlete undress’ that was reported by 27.6% of the coaches. Two items related to instrumental violence, ‘permitting or letting an athlete take part in a training or competition when they have had a medical recommendation not to participate’ and ‘forcing or asking an athlete to train when injured’, were witnessed by more than 35%, indicating that these kinds of behaviours are part of the sport culture. Percentages calculated for the items associated with physical violence showed that ‘throwing an object to an athlete’ was witnessed by 32.5% and ‘hitting an athlete with a hard object’ by 16% of the coaches (see detailed percentages for each item in Table 3).

**TABLE 3:** Valid *n* and percentages of witnessed IV behaviours towards athletes exhibited by coaches organised by IV type.

Items	*n*	Yes %	No %	Maybe %
Psychological violence				
Criticising an athlete excessively	400	70	22	8
Shouting insults, humiliating or making fun	399	64	28	8
Voluntarily rejecting or excluding an athlete	398	54	36	10
Voluntarily ignoring an athlete or be indifferent towards them	398	53	35	12
Instrumental violence				
Permitting or letting an athlete take part in a training or competition when they have had a medical recommendation not to participate	396	35	55	10
Forcing or asking an athlete to train when injured, when they had contrary medical advice	397	35	55	10
Forcing or asking an athlete to use fasting to reach the ideal weight in their sport	396	22	70	8
Asking an athlete to limit or restrict their contact with their social circle	393	19	76	5
Physical violence				
Throwing an object directly at an athlete	400	33	60	7
Hitting an athlete with a hand	399	28	66	6
Hitting an athlete with a hard object	399	16	78	6
Sexual violence				
Watching an athlete undress	395	28	68	4
Having a conversation of sexual nature with an athlete	395	9	90	1
Touching an athlete's nongenital areas	395	7	91	2
Having a sexual relation with an athlete through oral, vaginal or anal penetration	395	5	94	1
Watching an athlete masturbate	391	1	99	0.0

Coaches perceived physical violence as the most harmful form of IV towards athletes, followed closely by sexual, instrumental and lastly psychological violence which included neglect. Regarding their coaching approach, the mean score for empowering motivational climate was 4.43, suggesting that the coaches used behaviours that foster an environment promoting athlete autonomy, competence and a growth mindset. On the other hand, the mean score for disempowering motivational climate was 2.40, suggesting that on average, coaches made use of disempowerment behaviours in their coaching (see Table 4 for means and standard deviations).

**TABLE 4:** Means and standard deviations between perceived harmfulness of IV forms and coaching motivational climate.

Variables	*n*	*M*	SD
Harmfulness of physical IV	436	3.75	0.52
Harmfulness of psychological IV	436	3.56	0.60
Harmfulness of instrumental IV	434	3.68	0.52
Harmfulness of sexual IV	433	3.71	0.50
Empowering climate	474	4.43	0.42
Disempowering climate	474	2.40	0.61

### 3.2 Analysis by Subgroups

#### 3.2.1 Witnessing of IV Behaviours

Upon running chi‐square tests of independence, statistically significant associations (*p* < 0.05) were observed only on coaches' gender (men, women) related to the witnessing of five coach‐perpetrated IV behaviours suggestive of a pattern across psychological, physical and sexual forms of violence. Notably, women coaches were significantly more likely than men to report witnessing of: shouting insults, humiliating or making fun of athletes (χ^2^(2) = 7.09, *p* = 0.029); forcing or asking athletes to fast to reach an ideal weight (χ^2^(2) = 13.71, *p* = 0.001); forcing or asking an athlete to train when injured, contrary to medical advice (χ^2^(2) = 9.75, *p* = 0.008); engaging in a conversation of sexual nature with an athlete (χ^2^(2) = 6.66, *p* = 0.036); and having a sexual relationship with an athlete involving penetration (χ^2^(2) = 6.41, *p* = 0.040). In all these significant cases, the adjusted standardised residuals (ASRs ≥ |1.96|) highlighted the specific gender differences contributing to the overall association. Table 5 presents the chi‐square and ASR computations.

**TABLE 5:** Significant gender differences in witnessed interpersonal violence behaviours among coaches.

Witnessed variable…	*n*	*χ* ^*2*^	df	*p*	*Key Gender Difference*	*Interpretation*	*Strongest ASR (abs)*
Shouting insults, humiliating, or making fun	399	7.09	2	0.029	Women > Men (witnessed)	Women more likely to witness verbal abuse	2.6 (Men‐No)/2.0w(Women‐Yes)
Forcing or asking an athlete to fast for weight	396	13.71	2	0.001	Women > Men (witnessed)	Women more likely to witness weight control	3.7 (women‐Yes)/2.8 (Men‐No)
Forcing or asking an athlete to train when injured	397	9.75	2	0.008	Women > Men (witnessed)	Women more likely to witness unsafe training practices	2.7 (women‐Yes)/3.1 (Men‐No)
Conversation of sexual content with an athlete	395	6.66	2	0.036	Women > Men (witnessed)	Women more likely to witness sexual conversations	2.5 (Men‐No)
Sexual relation with an athlete	395	6.41	2	0.040	Women > Men (witnessed)	Women more likely to witness sexual relations	2.2 (women‐Yes)/2.5 (Men‐No)

*Note:* All listed items demonstrated statistically significant gender differences at *p* < 0.05. Positive ASRs indicate higher‐than‐expected frequencies; negative ASRs indicate fewer‐than‐expected frequencies.

Abbreviations: ASR, Adjusted standardised residual; *χ* ^*2*^ *,* Chi‐square value.

#### 3.2.2 Perceived Harmfulness of IV Behaviours

With regard to the forms of psychological and instrumental IV and perceived harmfulness of these, *t*‐test analysis showed a significant difference (*p* < 0.05) between men and women coaches. Women rated psychological (*p* = 0.001) and instrumental (*p* = 0.007) IV items higher than men, meaning they perceived it as more harmful. Based on the coach's professional status, a significant difference (*p* < 0.05) was found on sexual IV's perceived harmfulness. Paid coaches rated the items of sexual violence significantly higher than volunteer coaches (*p* = 0.019), though a small effect size was calculated. Subsequently, a significant difference with small effect sizes was also found between coaches with and without coaching certification on the perceived harmfulness of sexual violence (*p* = 0.033). Certified coaches perceived sexual forms as more harmful than coaches without coaching certification. No significant differences were found between perceived harmfulness and the level they were coaching. Table 6 presents the *t*‐test calculations and the perceived IV harmfulness variable.

**TABLE 6:** Independent *t*‐tests of coaches' characteristics and perceived harmfulness of IV behaviours.

		*n*	*M*(SD)	*t*(df)	*p*	*d*
	Coaches' gender					
Psychological violence^a^	Man	281	3.48 (0.66)	−3.23 (≈433)	0.001	−0.292
	Woman	154	3.68 (0.46)			
Instrumental violence^a^	Man	281	3.63 (0.56)	−2.73 (≈432)	0.007	−0.255
	Woman	152	3.76 (0.43)			
Physical violence	Man	281	3.74 (0.56)	−0.81 (434)	0.418	−0.080
	Woman	155	3.78 (0.43)			
Sexual violence	Man	280	3.68 (0.55)	−1.58 (431)	0.114	−0.160
	Woman	153	3.76 (0.39)			
	Professional status					
Psychological violence	Paid status	317	3.56 (0.60)	−0.07 (434)	0.943	−0.010
	Voluntary status	119	3.57 (0.61)			
Instrumental violence	Paid status	316	3.67 (0.52)	−0.58 (432)	0.561	−0.060
	Voluntary status	118	3.70 (0.51)			
Physical violence	Paid status	317	3.74 (0.51)	−0.47 (434)	0.641	−0.050
	Voluntary status	119	3.77 (0.52)			
Sexual violence	Paid status	316	3.74 (0.49)	2.361 (431)	0.019	0.260
	Voluntary status	117	3.62 (0.53)			
	Coaching certification					
Psychological violence	Not certified	52	3.45 (0.61)	−1.37 (417)	0.172	−0.200
	Certified	367	3.57 (0.61)			
Instrumental violence	Not certified	52	3.58 (0.58)	−1.32 (415)	0.188	−0.200
	Certified	365	3.68 (0.52)			
Physical violence	Not certified	52	3.60 (0.64)	−2.30 (417)	0.022	−0.340
	Certified	367	3.78 (0.49)			
Sexual violence	Not certified	52	3.56 (0.56)	−2.14 (414)	0.033	−0.317
	Certified	363	3.72 (0.50)			

^a^ Equal variances not assumed (Welch's test).

#### 3.2.3 Coaching Climate

Lastly, significant differences were found between men and women and coach's disempowering motivational climate. Men coaches significantly leaned to more disempowering motivational climates compared to women coaches (*Mp* = 0.020). However, it is important to note that the effect size was small. No other significant differences were found related to empowering and disempowering motivational climates and the other subgroups (see Table 6 for *t*‐test calculations).

#### 3.2.4 Associations Between Coaching Climate and IV Perceived Harmfulness

To examine the unique contributions of empowering and disempowering coaching climates to the perceived harmfulness of different forms of IV, both correlation and regression analyses were conducted. The results indicate that coaching climates play a role in shaping perceptions of harm, though the amount of explained variance in the regression models was relatively small in all cases.

Significant correlations were found between the coach's motivational climate and perceived harmfulness regarding all four forms of coach IV behaviours, and these are shown in Table 7. A positive correlation was observed between an empowering climate and perceived harmfulness across all IV behaviours, potentially indicating that coaches who perceive IV behaviours as more harmful also tend to foster an empowering coaching climate. Conversely, a negative correlation was found between perceived harmfulness and a disempowering climate, with coaches who perceive IV towards athletes as less harmful potentially fostering a more disempowering climate in their coaching.

**TABLE 7:** Correlations between perceived harmfulness of IV forms and coaching motivational climate.

Variables	*n*	Correlations				
		2	3	4	5	6
1. Harmfulness of physical IV	436	0.631^a^	0.636^a^	0.626^a^	0.104^b^	−0.136^a^
2. Harmfulness of psychological IV	436		0.610^a^	0.559^a^	0.142^a^	−0.262^a^
3. Harmfulness of instrumental IV	434			0.676^a^	0.197^a^	−0.183^a^
4. Harmfulness of sexual IV	433				0.125^b^	−0.170^a^
5. Empowering climate	474					−0.184^a^
6. Disempowering climate	474					

^a^ Correlation is significant at the 0.01 level (2‐tailed).

^b^ Correlation is significant at the 0.05 level (2‐tailed).

The multiple regression analyses further examined these relationships by assessing the unique contributions of empowering and disempowering climates to perceived harmfulness. Regression assumptions were tested; linearity and independence were met, homoscedasticity was acceptable (except for psychological violence), residuals showed minor deviations from normality typical of Likert data and predictors exhibited multicollinearity, which is acknowledged as a limitation. Missing data were handled by listwise deletion, with analyses conducted on the available cases. The models for all four types of IV were statistically significant (*p* < 0.01), reinforcing the role of coaching climates in shaping harm perceptions (see Table 8). However, the patterns of prediction varied by IV type: *Physical violence*: Only the disempowering coaching climate was a significant predictor, with a more disempowering climate associated with lower perceived harmfulness. The empowering climate did not contribute significantly. *Psychological violence*: Both empowering and disempowering climates were significant predictors. A more empowering climate was associated with greater perceived harmfulness, whereas a more disempowering climate was linked to lower perceived harmfulness. *Instrumental violence*: A similar pattern emerged, with both motivational climates playing a role. A more empowering climate was associated with higher perceived harmfulness, whereas a more disempowering climate was linked to lower perceived harm. *Sexual violence*: Only the disempowering coaching climate significantly predicted perceived harmfulness, with a more disempowering climate related to lower harm perception. The empowering climate did not have a significant effect.

**TABLE 8:** Regression analyses results predicting perceived harmfulness of IV forms from coaching motivational climates.

Dependent variable	*n*	*F*	*p*	*R* ^*2*^	Adjusted *R* ^*2*^	*B* (Empowering)	*p* (Empowering)	*B* (Disempowering)	*p* (Disempowering)
Physical violence	414	5.259	0.006	0.025	0.020	n.s.	n.s.	−0.101	0.015
Psychological violence	414	17.382	< 0.001	0.078	0.073	0.143	0.045	−0.242	< 0.001
Instrumental violence	413	13.375	< 0.001	0.061	0.057	0.218	< 0.001	−0.130	0.002
Sexual violence	412	8.041	< 0.001	0.038	0.033	n.s.	n.s.	−0.124	0.002

Abbreviation: B, Beta value.

## 4 Discussion

This study examined coaches' witnessing of IV behaviours exhibited by other coaches towards athletes, the perceived harm caused by these behaviours and the association between perceived harmfulness and the coaching climate fostered in Cyprus, Greece, Italy, Malta, Spain and Portugal. Psychological violence was the most frequently witnessed form of coach‐perpetrated IV, followed by instrumental, physical and sexual violence. However, physical violence was perceived as the most harmful, followed closely by sexual and instrumental violence, whereas psychological violence was viewed as the least harmful one. Most coaches reported that they foster an empowering climate, yet elements of a disempowering climate were present. Coaches who self‐reported fostering a more disempowering climate tended to perceive IV behaviours as less harmful than those fostering an empowering one. The findings indicate that coach‐perpetrated IV behaviours occur in the region characterised by varying perceptions of harmfulness and coaching climate. Both empowering and disempowering climates associate with perceived harmfulness of IV, particularly for psychological and instrumental violence. Nonetheless, the relatively low variance explained in all models suggests that additional factors contribute to perceptions harmfulness.

According to research inside and outside of sport, IV experiences can have severe consequences, including addiction, depression, suicide, school dropout, unemployment and relationship difficulties (e.g., Parent et al. 2022; Vertommen et al. 2018; Willson et al. 2023; World Health Organization 2014). However, psychological violence in sport, like verbal aggression, is commonly normalised as a ‘motivational tool’ (Gervis and Dunn 2004; Stirling 2013; McGee et al. 2024), particularly in high‐performance settings where toughness is a desirable trait. This may explain why coaches in this study perceived psychological violence as the least harmful while witnessing it frequently. The occurrence of psychological violence has been linked to win‐focused coaching cultures, where shouting, humiliating and ridiculing athletes are mistakenly believed to enhance performance (Kelly and Waddington 2006; Roberts et al. 2020). Although physical violence was less frequently witnessed, it was considered the most harmful. We understand that while coaches may be aware of IV's negative impact, cultural factors may also influence the normalisation and tolerance of IV behaviours. Cultural beliefs and practices in Mediterranean sport, like the minimisation of sexual abuse, the normalisation of touching and of controlling coach–athlete relationships reported for instance in Cyprus, Greece and Portugal (see, Alexandre et al. 2022; Chroni and Kavoura 2022; Papaefstathiou 2014) may account for the perception that physical violence is more harmful than other forms, such as sexual violence. Especially given that, in the Greek general population, emotional abuse in intimate relationships is relatively common, yet physical abuse is more readily recognised and more often linked to serious personal consequences (Papadakaki et al. 2009). Future research should explore acceptance levels of IV behaviours across national sports models that emphasise athlete development over results, considering that the normalisation of abuse in sports can be reinforced by deeply ingrained cultural norms and organisational structures that enable violence (McGee et al. 2024). Psychological abuse, in particular, is perpetuated by athletes' over‐conformity to the sport ethic, making it appear as an accepted, even beneficial, aspect of coaching rather than a violation (Fournier et al. 2021; Kavanagh et al. 2017). Over‐conformity to the sport ethic refers to athletes unquestioningly adopting socialised norms and engaging in behaviours that prioritise performance above all else, often at the expense of their well‐being (McGee et al. 2025). Gender biases and traditional norms contribute to this culture, where harassment is often overlooked or excused (Berger et al. 2019).

The coaches' gender revealed further differences in witnessing and perceiving harm caused by IV behaviours. Women significantly more than men coaches were likely to report witnessing verbal humiliation, coercive weight‐control practices, pressures on injured athletes to train and even sexual misconduct. In contrast, men were more likely to report not witnessing these, possibly reflecting their lack of awareness, desensitisation, or denial that characterises cultures of silence in the Mediterranean (Chroni et al. 2023). Overall, women coaches consistently perceived psychological and instrumental forms of IV as more harmful than men. This gendered pattern of greater sensitivity to abuse and lower tolerance for harmful behaviours among women coaches aligns with findings that being a girl or a woman is associated with higher odds of reporting interpersonal violence experiences (Parent and Vaillancourt‐Morel's 2021; Zogg et al. 2024) work who found that being a girl/woman was related to higher odds of reporting IV experience. These findings highlight the need for more research exploring how gender differences in recognising IV behaviours and perceiving these as more/less harmful are influenced by cultural factors, coach education or the professionalisation of coaches and coaching, sport type, age or performance level as well. Furthermore, paid coaches and educated ones rated sexual violence as more harmful than volunteers and uneducated coaches, suggesting that coaches' education and recruitment and hiring criteria for them need apt attention. Of course, the small effect sizes we calculated indicate that although these characteristics play a role, they may interact with other systemic and cultural dynamics, not explored here.

Looking at the coaching climate, although most of our coaches aimed to create an empowering one supportive of athlete autonomy, growth and well‐being, some did incorporate elements of disempowering climate, like controlling and negative behaviours that undermine autonomy, confidence and well‐being. These findings also underscore the need for coaching education that promotes empowering practices and raises awareness on how coaches' approaches may unintentionally enable harmful behaviours. Existing research highlights the importance of fostering a supportive environment to enhance athlete safety while addressing the potential harm of various forms of violence (Birr et al. 2023; Greither and Ohlert 2023; Ohlert et al. 2022). Our findings reinforce the need for mandatory, culturally tailored education programmes on safe coaching, echoing recent calls for evidence‐based training grounded in coaches' expressed needs (Gillard et al. 2025). Findings particularly indicate that coaching climate influences perceived IV harmfulness. The empowering climate is associated with greater perceived harm—particularly in psychological and instrumental violence—though the relationships were somewhat weak. The disempowering climate correlated with lower perceived harm and showed weak associations with higher perceived harmfulness across all IV forms. Analyses confirmed that both coaching climates predicted IV harm perception, but with low explanatory power, suggesting the influence of additional individual and/or systemic factors (see Kerr et al. 2019; Mountjoy et al. 2016). Future research should consider how elements like personal experiences, exposure to ethics, duty‐of‐care, compassion, sport‐specific norms and organisational policies interact with the coaching climate to shape perceptions of IV harm, particularly in the Mediterranean region.

These factors likely interact in complex ways beyond what this study captured. Empowering coaching climate aligns with self‐determination theory (Deci and Ryan 2000), which emphasises autonomy, competence and relatedness. Such climate may heighten sensitivity to IV harm by promoting athlete‐centred development and positive coach–athlete relationships (Jowett 2017). In contrast, the disempowering climate, often characterised by controlling behaviours, may reinforce hierarchical and punitive norms, leading to lower IV perceived harmfulness (Duda et al. 2017). These findings and Santos et al. (2013) work, where Portuguese elite coaches appeared to promote an illusion of empowerment while maintaining strong hierarchical control aiming to generate compliance and respect, indicate that coaches know right from wrong. Future research should look how societal and sport cultures influence coaches' rationale in the orchestration of such behaviours.

Concerning disempowering climate, gender appeared to be more relevant than other demographic and professional characteristics in relation to disempowering coaching behaviours, with men coaches employing it more than women. Schyvinck et al. (2025) and Zogg et al. (2024) found controlling coaching and nonautonomy‐supportive coaching, respectively, to be associated with athlete experiences of IV. Mediterranean coaching cultures are characterised by hierarchical relationships, traditional gender roles and blurred boundaries that suppress autonomy (Chroni and Kavoura 2022; Chroni et al. 2023; Santos et al. 2013), which possibly contribute to the endorsement of disempowering climate and lower IV harm perceptions. In settings where IV is seen as a functional approach for motivating and developing athletes, dominative authoritative behaviours appear to be more readily learnt and accepted (Roberts et al. 2020).

The study's findings reveal an urgent need for safe sport training of North Mediterranean coaches. Governing bodies should integrate (or revisit) ethics and violence prevention modules into coaching certification programmes and offer continuous education courses. Practical recommendations for improving coach education and policy include: (i) Mandatory safe sport training to help coaches recognise IV behaviours commonly going unnoticed due to cultural stereotypes and having been normalised in their sport and society. (ii) Interventions addressing how to replace verbal aggressions and coercion practices with empowering ones that motivate and prioritise the athlete and their well‐being. (iii) Gender‐sensitive safe sport training addressing gender typical norms and beliefs (e.g., (sexual) violence, practice of consent, etc.), with tailored messaging for men coaches.

A key strength of this study is its broad examination of how coaching climates associate with perceptions of harmfulness, contributing to better recognition of cultural influences on IV in sport. However, limitations include reliance on self‐reported data, the relatively low variance explained by coaching climate and the low reliability of the disempowering scale, which we retained as internal consistency estimates in cross‐cultural research can vary, particularly for constructs that are broad and multidimensional (Van de Vijver and Hambleton 1996). We also did not conduct a confirmatory factor analysis of the adapted 16‐item version of the VTAQ, meaning that the factorial validity of this abbreviated, culture‐sensitive item set cannot be fully established here. Future research should examine the structural validity of shortened and culturally adapted IV measures in larger samples before they are used more widely. In addition, a limitation of the regression analyses is the high multicollinearity between empowering and disempowering coaching climates, which reduces the precision of estimating their unique effects. This overlap is theoretically expected, as both constructs stem from the same motivational climate framework, but it should be noted that the shared variance makes it difficult to isolate their independent contributions. Nevertheless, the overall pattern of associations across the models provides meaningful evidence that coaching climates are related to athletes' harm perceptions (see Birr et al. 2023; Greither and Ohlert 2023; Ohlert et al. 2022). Hence, the findings should be interpreted with caution.

A further set of limitations concerns measurement choices around IV behaviours. First, some items combine different degrees of agency (e.g., ‘forcing or asking an athlete to train when injured’), which may blur distinctions between clearly coercive practices and more ambiguous situations in which athletes feel they can refuse. Within the psychological violence items, behaviours also ranged from more subtle actions (e.g., ignoring an athlete) to overtly hostile acts (e.g., humiliating an athlete), which may not be perceived as equally severe and could introduce additional heterogeneity into this subscale. Second, the witnessing question (‘Have you ever witnessed this behaviour?’ with yes/no/maybe responses) did not capture when, how often or in which context coaches observed these behaviours, nor did it distinguish between direct observation and second‐hand accounts. As such, our prevalence estimates cannot be interpreted as precise indicators of the current frequency of IV in sport, but rather as indicating whether coaches have, at some point in their careers, encountered these behaviours. Third, considering that IV behaviours may occur in all countries, sports and levels, we did not address coaches' witnessing by sport, age group or performance level, all of which may shape what they see and how they respond. These issues should be addressed in future research through more fine‐grained and context‐specific assessment strategies.

In closing, the study underscores the need for the research‐based culture‐sensitive approach to addressing IV in sport. For instance, although empowering coaching aligned with seeing more harm in IV, still, the coach's gender, professional status and education made a difference. For the six Mediterranean countries of the study and other ones of similar cultural characteristics, the findings call for interventions that will aptly challenge the normalisation of psychological (and other forms of) violence, increase awareness and capacity to recognise IV (possibly in contradiction to social norms and traditions) and equip the coaches with a toolbox that helps them foster safe, supportive and thriving sport environments.

## Conflicts of Interest

The authors declare no conflicts of interest.
